# Fifty‐year history of biliary surgery

**DOI:** 10.1002/ags3.12289

**Published:** 2019-09-30

**Authors:** Masato Nagino

**Affiliations:** ^1^ Division of Surgical Oncology Department of Surgery Nagoya University Graduate School of Medicine Nagoya Japan

**Keywords:** biliary surgery, biliary tract cancer, extended hepatectomy, hepatopancreatoduodenectomy, vascular resection

## Abstract

There has been enormous progress in the surgical treatment of biliary tract cancers in the past 50 years. In preoperative management, biliary drainage methods have changed from percutaneous transhepatic biliary drainage to endoscopic nasobiliary drainage, while the advent of multidetector‐row computed tomography in imaging diagnostics now enables visualization of three‐dimensional anatomy, extent of cancer progression, and hepatic segment volume. Portal vein embolization has also greatly improved the safety of extended hepatectomy, and indication of extended hepatectomy can now be objectively determined with a combination of the indocyanine green test and computed tomography volumetry. In terms of surgery, combined resection and reconstruction of the portal vein and/or hepatic artery can now be safely carried out at specialized centers. Further, long‐term survival can be attained with combined vascular resection if R0 resection can be achieved, even in locally advanced cancer. Hepatopancreatoduodenectomy, combined major hepatectomy with pancreatoduodenectomy, should be aggressively carried out for laterally advanced cholangiocarcinoma, whereas its indication for advanced gallbladder cancer should be carefully evaluated. Japanese surgeons have made a significant contribution to the progression of extended surgeries such as combined vascular resection and hepatopancreatoduodenectomy for biliary tract cancer.

## Abstract 2

Changes in preoperative management of hepatectomy for biliary tract cancer.

## Graphical abstract

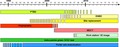

## 1 INTRODUCTION

Diseases treated by biliary surgery are broadly divided into biliary tract cancers such as cholangiocarcinoma, gallbladder cancer, and carcinoma of Vater's ampulla and benign diseases such as cholelithiasis, bile duct injury, and postoperative bile duct stricture. As a result of the limited literature available, this article will review the history of surgical treatments for biliary tract cancers, focusing on preoperative management and extended surgical procedures in particular.

## 2 CHANGES IN PREOPERATIVE MANAGEMENT OF HEPATECTOMY FOR BILIARY TRACT CANCERS

### 2.1 Preoperative biliary drainage

**Figure 1:**
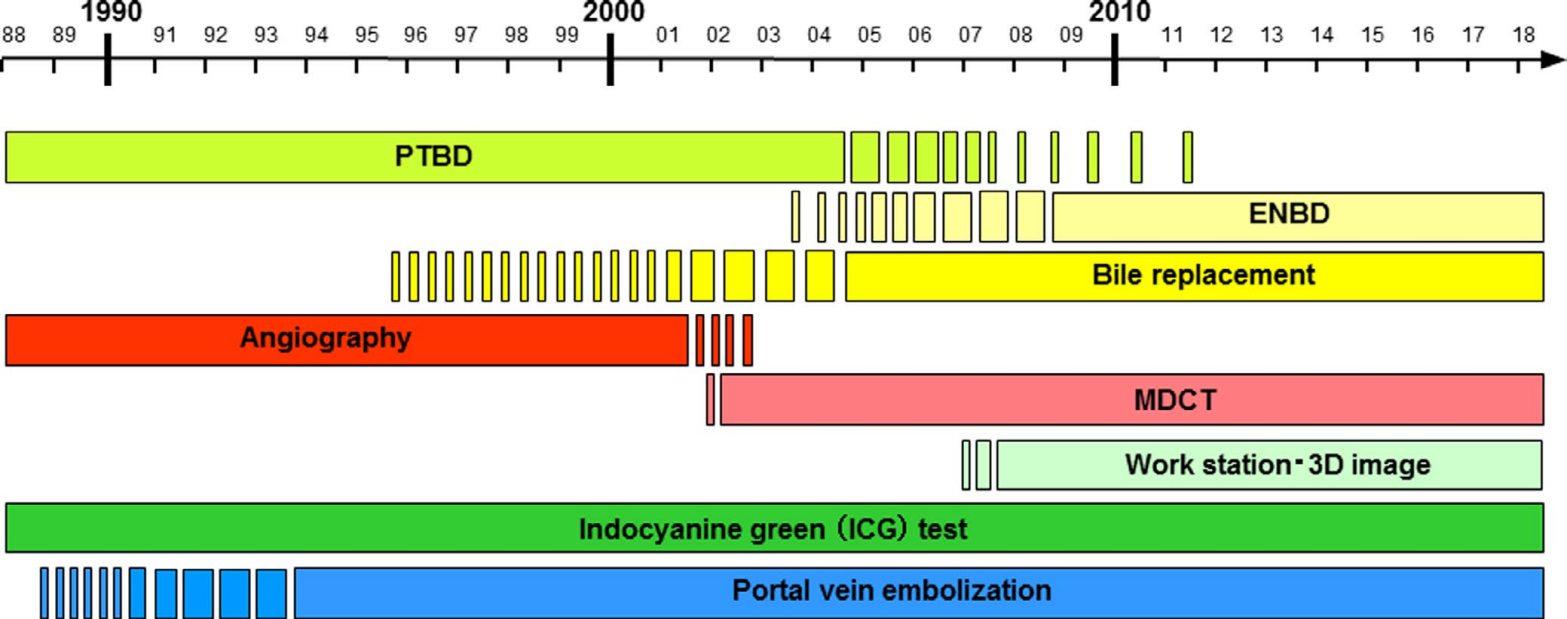
Changes in preoperative management of hepatectomy for biliary tract cancer. ENBD, endoscopic nasobiliary drainage; MDCT, multidetector‐row computed tomography; PTBD, percutaneous transhepatic biliary drainage

In the 1980s, a number of randomized controlled trials (RCT) were conducted in Western countries on the clinical value of percutaneous transhepatic biliary drainage (PTBD).^1^, ^2^, ^3^ Results of the RCT showed that preoperative drainage with PTBD had no favorable effects on surgical outcome, with no advantages in terms of cost. However, the majority of the study cases involved palliative resections including bypass surgery, and only a small number of hepatectomy cases for biliary tract cancers were included. Furthermore, in many of the RCT, the incidence of complications caused by PTBD itself was extremely high, and drainage duration was insufficient, so Japanese surgeons did not accept these results. In Japan, preoperative biliary drainage with PTBD was widely implemented until approximately 2010 (Figure 1). However, PTBD was found to cause so‐called seeding metastasis, including sinus tract recurrence,^4^ peritoneal dissemination,^5^, ^6^ or pleural dissemination;^7^ thus, endoscopic nasobiliary drainage (ENBD) is commonly carried out as preoperative drainage before extended hepatectomy for biliary cancer.^8^, ^9^, ^10^ Now, ENBD is recommended as first‐line treatment in Japanese clinical practice guidelines for the management of biliary tract cancers.^11^ As ENBD is external drainage where the drained bile is eliminated out of the body, it is recommended to return the bile to the intestinal tract.^10^, ^12^ ENBD is a superior method with few cholangitis complications, but it does present pharyngeal discomfort,^13^ so, in the future, it is highly likely to be replaced with an inside stent.^14^

### 2.2 Preoperative diagnostic modality

Prior to 2000, along with direct cholangiography with percutaneous transhepatic cholangiography (PTC)/PTBD, abdominal angiography was also widely used to ascertain the anatomy of the hepatic artery (HA) and portal vein (PV) and to diagnose the extent of cancer as a preoperative diagnostic imaging modality (1). The advent of multidetector‐row computed tomography (MDCT) in the 2000s drastically changed preoperative imaging diagnostics in the field of hepatobiliary‐pancreatic medicine. MDCT used in combination with a workstation for image analysis easily generates a great deal of information within 1‐2 hours after taking the CT scan, including diagnosis of the extent of cancer;^15^, ^16^, ^17^ diagnosis of distant metastases in areas such as the peritoneum, liver, or lymph nodes,^18^D structure of the HA, PV and hepatic vein,^19^, ^20^, ^21^, ^22^ and volume of the hepatic segments.^21^, ^22^, ^23^ Consequently, abdominal angiography was never implemented. In many cases, the resection procedure could be planned based on MDCT information alone. In our clinic, MDCT is routinely carried out on the day of admission and, based on this information, the site of biliary drainage is determined and portal vein embolization is scheduled. Magnetic resonance imaging (MRI) and positron emission tomography are used only for selected patients.

Around the time when PTBD was widely implemented, biopsy for biliary tract cancer was done by percutaneous transhepatic cholangioscopy (PTCS).^24^ However, as mentioned above, due to concerns about seeding metastasis associated with PTBD,^4^, ^5^, ^6^, ^7^ PTCS is not used for preoperative biliary biopsy. Instead, an endoscopic transpapillary approach is now commonly used.^25^ Externally drained bile has been used for bile cytology, but the accuracy of this method is modest, at approximately 50%.^26^, ^27^ Unlike stomach cancer and colon cancer, it is difficult to repeatedly take sufficient biopsy samples for bile duct cancers. Thus, surgery is often carried out when cancer is strongly suspected based on imaging findings, even without preoperative histological confirmation. Consequently, approximately 3% of cases resected as perihilar cholangiocarcinoma are so‐called misdiagnoses, where the lesion is diagnosed as benign in final pathology.^28^

### 2.3 Liver function assessment and portal vein embolization

Loading test using indocyanine green (ICG) has traditionally been used in Japan as preoperative liver function tests for over 30 years. Asialoscintigraphy and galactose tolerance tests are also used as liver function tests. However, there are no methods superior to the ICG test in terms of simplicity and reliability, and this test is still routinely carried out before hepatectomy (Figure 1). The most important aspect from the perspective of clinical surgery is accurately predicting the extent of resection rate based on liver function, prior to surgery. From this viewpoint, different authors^29^, ^30^, ^31^ proposed hepatectomy criteria incorporating evaluation of hepatic reserve based on the ICG test. These hepatectomy criteria were devised mainly from analysis of hepatectomy cases for hepatocellular carcinoma from over 20 years ago. Nonetheless, all are excellent criteria, and some are still currently in use in medical institutions. Our group has examined many hepatectomy cases with biliary tract cancers and has reported hepatectomy criteria based on future liver remnant plasma clearance rate of indocyanine green (ICGK‐F) values calculated by multiplying the ICGK value by the residual liver volume ratio.^32^, ^33^, ^34^ This method is simple and is used in a large number of medical institutions.^10^

Portal vein embolization (PVE) is an excellent method reported by Makuuchi et al as a preoperative procedure for safely carrying out extended hepatectomy.^35^, ^36^ The first case was done on June 8, 1982, on a patient with gallbladder cancer. Embolization of the right PV increases the volume of the left lobe by approximately 10% in 2‐3 weeks.^32^, ^37^, ^38^, ^39^ and immediately reduces the resection rate by 10%, thereby mitigating the risk of extended hepatectomy. Nagino et al from Nagoya University developed the ipsilateral approach as a PVE technique^40^, ^41^ and reported right trisegment and left trisegment PVE for the first time using this technique.^42^, ^43^ The ipsilateral approach is safer than the conventional contralateral approach,^35^, ^36^, ^44^ and now PVE using this technique is carried out worldwide. Recently, some medical institutions use associated liver partition and PV ligation in staged hepatectomy (ALPPS) for perihilar cholangiocarcinoma to increase the volume of the planned residual liver;^45^ however, the surgical outcomes after ALPPS are extremely poor, and this technique should not be used in surgery for Klatskin tumor.^46^

## 3 CHANGES IN EXTENDED SURGERY FOR BILIARY TRACT CANCERS

Hepatectomy combined with vascular resection (combined resection of the PV and/or HA) and hepatopancreatoduodenectomy (HPD) are proposed as extended surgery for biliary tract cancers. Below, the changes in these procedures are described.

### 3.1 Hepatectomy with combined vascular resection

Doctor Kajitani from the Cancer Institute Hospital carried out the world's first hepatectomy with PV resection for perihilar cholangiocarcinoma on August 6, 1965.^47^ He resected the right hepatic lobe, but not the caudate lobe. The PV including portal bifurcation was resected and reconstructed by anastomosis between the upstream side of the PV and the inferior vena cava in an end‐to‐side method (Eck fistula). Operative time was 4 hours and 2 minutes, and blood loss was 4300 g. The patient developed no liver failure despite the Eck fistula and was discharged in good health, but died of cancer recurrence 3 years and 11 months later. Thereafter, until around 1990, Longmire,^48^ Fortner,^49^ Tsuzuki,^50^ Blumgart,^51^ and Sakaguchi,^52^ and respective colleagues reported their surgical experience with hepatectomy with PV resection, but there were only a few cases in each study. In 1981, Tsuzuki et al from Keio University reported two cases of left hepatectomy with simultaneous resection of the PV and HA.^50^ Both patients tolerated the procedure but died of cancer recurrence at 1 year and 6 months and at 1 year and 3 months later, respectively. Nonetheless, these were the world's first successful cases of simultaneous resection of the PV and HA, and represented a groundbreaking report (Table 1).

**Table 1:** Reports on hepatectomy with vascular resection for biliary cancer

Year	First author	Country	Procedure	Comment
1965	Kajitani^47^	Japan	Right Hx + PV (n = 1)	First successful case of Hx + PV
1973	Longmire^48^	USA	Right trisectionectomy + PV (n = 2)	Survived
1974	Fortner^49^	USA	Major Hx + PV (n = 3)	All dead
1983	Tsuzuki^50^	Japan	Left Hx + PV･HA (n = 2)	First successful case of Hx + PV･HA
1984	Blumgart^51^	UK	Major Hx + PV (n = 3)	Survived
1986	Sakaguchi^52^	Japan	Right trisectionectomy + PV (n = 8)	Introduction of “insert anastomosis”, 1 dead,
1991	Nimura^53^	Japan	Major Hx + PV (n = 29)	First large series, mortality = 17%, 3‐/5‐y survival = 29%/6%
1993	Tashiro^54^	Japan	Major Hx + PV (n = 6)	All survived, R0 resection (n = 2)
1994	Sugiura^55^	Japan	Major Hx + PV (n = 18)/HA (n = 4)	Keio multicenter study
1996	Pichlmayr^56^	Germany	Major Hx + PV (n = 33), HA (n = 1), PV･HA (n = 2)	Comparison between Hx and liver transplantation
1997	Miyazaki^57^	Japan	Major Hx + PV (n = 34)	Use of left renal vein graft (n = 4)
1999	Neuhaus^58^	Germany	Major Hx + PV (n = 23)	Mortality = 17%, right trisectionectomy + PV is recommended
2000	Lee^59^	South Korea	Major Hx + PV (n = 29), HA (n = 4)	Mortality = 13.3%, use of external iliac vein graft
2001	Yamanaka^60^	Japan	Right or left Hx + PV (n = 5), HA (n = 3), PV･HA (n = 7)	Mortality = 8%, microsurgical technique is useful
2003	Ebata^61^	Japan	Major Hx + PV (n = 52)	Mortality = 9.6%, 5‐y survival = 9.9%
2006	Shimada^62^	Japan	Major Hx + PV (n = 3), HA (n = 6), PV･HA (n = 6)	Mortality = 13.3%, vascular resection for GBC is not justified
2006	Sakamoto^63^	Japan	Left‐sided or central Hx + HA (n = 11)	Mortality = 0%, HA can be safely carried out
2006	Hemming^64^	USA	Major Hx + PV (n = 26)	Mortality = 4%, 5‐y survival = 39%
2007	Miyazaki^65^	Japan	Major Hx + PV (n = 34), HA (n = 2), PV･HA (n = 7)	3‐y survival of HA or HA･PV = 0%. HA is not justified
2010	Nagino^66^	Japan	Major Hx + PV･HA (n = 50)	Mortality = 2%, 5‐y survival = 30%, PV･HA is justified
2016	Matsuyama^67^	Japan	Major Hx + PV (n = 54), HA (n = 44)	Mortality = 6.1%, 5‐y survival = 51% (PV), 22% (HA)

Abbreviations: GBC, gallbladder cancer; HA, hepatic artery resection; Hx, hepatectomy; PV, portal vein resection; PV･HA, simultaneous resection of portal vein and hepatic artery.

By the 1990s, reports appeared on combined vascular resections for over 20 patients.^53^, ^55^, ^56^, ^57^, ^58^ In 1991, Nimura et al from Nagoya University described surgical outcomes of 29 cases of hepatectomy with PV resection for locally advanced biliary tract cancers; this was the first large series study on combined PV resection for biliary tract cancer.^53^ Surgical mortality rate was 17.2%, and the 3‐ and 5‐year survival rates were 29% and 6%, respectively. In 1997, Miyazaki et al from Chiba University reported use of the left renal vein graft for long PV resections that required graft reconstruction.^57^ Harvesting the left renal vein is simple, and this procedure is still used today as an option for vein grafts. In 1999, Neuhaus et al reported outcomes of hepatectomy with PV resection for perihilar cholangiocarcinoma (n = 23), emphasizing that right trisectionectomy with PV resection had the best curability rate.^58^ Thereafter, they reported the efficacy of a surgical technique that routinely combined en bloc resection of the PV with right trisectionectomy, known as the “No‐touch technique”.^68^ This technique was once adopted by some medical institutions in Japan;^69^ however, due to doubts about the surgical oncological significance of this procedure, at present, there are no medical institutions in Japan that use the No‐touch technique for perihilar cholangiocarcinoma.

From 2000 onwards, reports appeared on highly difficult hepatectomy with HA resection.^59^, ^60^, ^62^, ^63^, ^65^, ^66^, ^67^ In 2007, Miyazaki et al reported that short‐term outcomes of PV resection for perihilar cholangiocarcinoma were within the acceptable range, and there were some long‐term survivors; however, they reported that HA resection could not be justified as a result of the high mortality rate and the absence of long‐term survivors beyond 3 years.^65^ In 2010, Nagino et al reported the outcomes of 50 patients treated with the most difficult procedure for perihilar cholangiocarcinoma, hepatectomy with simultaneous resection of the PV and HA.^66^ Types of hepatectomy carried out included left trisectionectomy with caudate lobectomy (n = 26), left hepatectomy with caudate lobectomy (n = 23), and right hepatectomy with caudate lobectomy (n = 1). The mortality rate was low (2%), and the 5‐year survival rate was unexpectedly better (30%). These findings proved that if R0 resection was achievable with extended surgery, then long‐term survival was attainable even with locally advanced cancer, which was previously considered inoperable. Matsuyama et al from Yokohama City University also reported that four out of 44 patients survived 5 years after HA resection for perihilar cholangiocarcinoma.^67^

### 3.2 Major hepatopancreatoduodenectomy

Major HPD, which combines major hepatectomy with pancreatoduodenectomy, is the most difficult surgical procedure. The world's first case of major HPD was carried out on June 12, 1974, at the Cancer Institute Hospital for a bulky advanced gallbladder cancer involving the duodenum. The surgeon was Dr Kuno, the chief surgeon of the hospital. Operative time was 6 hours and 25 minutes, and blood loss was 3270 mL. The patient was discharged after 2 months but died of cancer recurrence 5 months postoperatively. Kasumi et al gave a brief report of this case,^70^ whereas the first detailed report on major HPD was written by Takasaki et al from Tokyo Woman's Medical School.^71^ They carried out major HPD on five patients with advanced gallbladder cancer. All patients underwent extended right hepatectomy, and all surgeries were advanced in a PD‐first method.^71^ Unfortunately, three of the five patients died of postoperative complications, while the remaining two patients survived recurrence‐free for 16 months and 5 months, respectively. In the 1980s, major HPD was resolutely carried out by Japanese surgeons mainly for advanced gallbladder cancer, but the mortality rate was high and prognosis was poor (Table 2).^70^, ^71^, ^72^, ^73^, ^74^, ^75^ At that time, major HPD was not carried out outside of Japan, so the valuable and challenging achievements by Japanese surgeons in the early days of major HPD were all written in Japanese‐language literature,^70^, ^71^, ^72^, ^73^, ^74^, ^75^ and it is extremely regrettable that these reports were not communicated to the rest of the world.

**Table 2:** Initial reports on major HPD for advanced biliary cancer by Japanese surgeons

Year	First author	Disease	No. of HPD	No. of portal vein resections	Mortality
1976	Kasumi^70^	GBC	1	0	0
1980	Takasaki^71^	GBC	5	0	3 (60%)
1983	Nakamura^72^	GBC	2	1	0
1987	Sugiura^73^	GBC	16	7	6 (38%)
1987	Nimura^74^	GBC	10	5	2 (20%)
1988	Hanyu^75^	GBC	3	3	1 (33%)

Note

Note that all of the above six reports were written in Japanese.

Abbreviations: GBC, gallbladder cancer; HPD, hepatopancreatoduodenectomy.

Several reports have been written since 2000 (3), and the mortality rate has fallen below 20%.^76^, ^77^, ^78^, ^79^, ^80^, ^81^, ^82^, ^83^, ^84^, ^85^, ^86^ An important finding identified in these reports was that, although major HPD could achieve good prognosis for cholangiocarcinoma, there was no improvement in prognosis when major HPD was carried out for advanced gallbladder cancer. Ebata et al from Nagoya University reported on the outcome of HPD for 85 cases of cholangiocarcinoma and found the mortality rate was low at 2% and the 5‐year survival rate for all resected patients was 37%.^81^ The long‐term outcomes were extremely good with 5‐year survival of 54% in 57 patients who underwent R0 resection, with no distant metastasis.^81^ Thus, they emphasized the clinical value of proactively implementing HPD for laterally advanced cholangiocarcinoma. Conversely, Kaneoka et al from Ogaki Municipal Hospital^78^ and Sakamoto et al from National Cancer Center^83^ reported that no patients who underwent major HPD for advanced gallbladder cancer survived after 5 years. Aoki et al from Tokyo University found no significant differences in the prognosis of gallbladder cancer and cholangiocarcinoma after HPD;^85^ however, it should be noted that six of the 13 patients with gallbladder cancer underwent small hepatectomy including liver bed resection. Recently, Mizuno et al from Nagoya University reported the outcome of major HPD for 38 patients with gallbladder cancer,^86^ the largest series to date, but they found the mortality rate remained high at 18%, and the 5‐year survival for all patients who underwent major HPD was poor at 11%. In their series, three patients who survived for longer than 5 years had cystic duct cancer. There were no long‐term survivors among patients with advanced gallbladder cancer involving the hepatoduodenal ligament and/or pancreas that required major HPD. Therefore, the authors mentioned that upfront surgery is not indicated for such advanced gallbladder cancer and, instead, it is recommended to first carry out chemotherapy, then reassess the patient's condition before deciding on resection.^86^ Indication for major HPD should be considered separately for cholangiocarcinoma and gallbladder cancer.

**Table 3:**
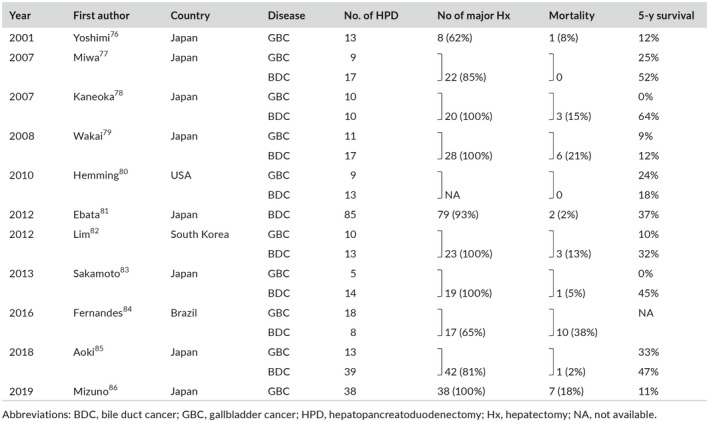
Reports on major HPD for advanced biliary cancer after 2000

Although not mentioned in detail, it is evident that reduction of intraoperative blood loss has greatly contributed to the improvement of the safety of hepatobiliary surgery.^87^ Extended hepatobiliary resections presented here are still associated with much intraoperative blood loss; thus, further reduction of blood loss is key to further improve surgical outcome after extended resection.

## 4 CLOSING REMARKS

**Figure 2:**
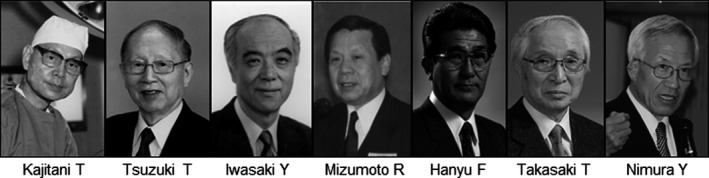
Japanese pioneers in biliary surgery

In all modesty, undoubtedly Japanese surgeons (Figure 2) have made significant contributions to the progression of biliary surgery, particularly difficult extended surgery for biliary tract cancers. Hepatectomy with PV resection,^47^ hepatectomy with simultaneous resection of the PV and HA,^50^ and major HPD,^70^ all of which are still demanding to carry out, were successfully done for the first time by Japanese surgeons. We thus have great pride in these achievements. Japanese surgeons may be suited to surgical treatment of biliary tract cancers, which require careful pre‐ and postoperative management and meticulous surgical techniques. Although we should express respect for the pioneers of these techniques, we must also strongly encourage further developments in biliary surgery.

## DISCLOSURE

Conflicts of Interest: Author declares no conflicts of interest for this article.
